# An Outbreak of Dengue Fever in St. Croix (US Virgin Islands), 2005

**DOI:** 10.1371/journal.pone.0013729

**Published:** 2010-10-28

**Authors:** Hamish Mohammed, Mary Ramos, Julie Armstrong, Jorge Muñoz-Jordán, Kathleen O. Arnold-Lewis, Aurimar Ayala, Gary G. Clark, Eugene S. Tull, Mark E. Beatty

**Affiliations:** 1 Dengue Branch, Division of Vector-Borne Infectious Diseases, Centers for Disease Control and Prevention, San Juan, Puerto Rico; 2 Ross University School of Veterinary Medicine, St. Kitts, West Indies; 3 Department of Pediatrics, University of New Mexico, Albuquerque, New Mexico, United States of America; 4 United States Virgin Islands Department of Health, St. Croix, United States Virgin Islands; 5 Office of the Director, Centers for Disease Control and Prevention, Atlanta, Georgia, United States of America; 6 Governor Juan F. Luis Hospital, St. Croix, United States Virgin Islands; 7 Mosquito and Fly Research Unit, United States Department of Agriculture, Gainesville, Florida, United States of America; 8 Pediatric Dengue Vaccine Initiative, Seoul, South Korea; University of Cape Town, South Africa

## Abstract

**Background:**

Periodic outbreaks of dengue fever occur in the United States Virgin Islands. In June 2005, an outbreak of dengue virus (DENV) serotype-2 with cases of dengue hemorrhagic fever (DHF) was detected in St. Croix, US Virgin Islands. The objective of this report is to describe this outbreak of DENV-2 and the findings of a case-control study examining risk factors for DHF.

**Methodology/Principal Findings:**

This is the largest dengue outbreak ever recorded in St. Croix, with 331 suspected dengue cases reported island-wide during 2005 (62.2 cases/10,000 population); 54% were hospitalized, 21% had at least one hemorrhagic manifestation, 28% had thrombocytopenia, 5% had DHF and 1 patient died. Eighty-nine laboratory-positive hospitalized patients were identified. Of these, there were 15 (17%) who met the WHO criteria for DHF (cases) and 74 (83%) who did not (controls). The only variable significantly associated with DHF on bivariate or multivariable analysis was age, with an adjusted odds ratio (95% confidence interval) of 1.033 (1.003,1.064).

**Conclusions/Significance:**

During this outbreak of DENV-2, a high proportion of cases developed DHF and increasing age was significantly associated with DHF.

## Introduction

Dengue fever (DF) is an acute infection caused by any of the four serotypes of dengue virus (DENV-1, -2, -3 and -4). In the Americas, the virus is transmitted by *Aedes aegypti* mosquitoes. Infection confers transient cross-immunity to all serotypes and lifelong immunity to the infecting serotype. Each of the four serotypes can cause DF or the potentially fatal clinical syndrome of dengue hemorrhagic fever (DHF), characterized by acute febrile illness with thrombocytopenia, hemorrhage, and capillary leakage. [1] Although the pathogenesis of DHF is not fully understood, the most important risk factor is a prior dengue infection. [2] Viral strain and host factors also play a role. [3], [4]

St. Croix, a Caribbean island and the largest (land area of 218 km^2^) of the four inhabited United States Virgin Islands (USVI — St. Croix, St. Thomas, St. John, and Water Island), is located approximately 60 miles east of Puerto Rico and has a population of 53,234. [5] The racial and ethnic distribution is 73% Afro-Caribbean and 27% other/mixed races — 21% of the population self-identifies as Hispanic or Latino. [5] There are periodic outbreaks of DENV in the USVI, [6] and DENV-1, -2 and -4 have been detected on separate occasions from 1981 to 1996. [7], [8] Prior to 2005, the most recently documented dengue outbreak occurred in October 2004 in St. Thomas, when over 40 cases were reported (CDC, unpublished data).

In June 2005, the Virgin Islands Department of Health (VIDOH) detected an increase in reported DF cases from St. Croix, and invited the DB to assist with the outbreak investigation. The objective of this manuscript is to describe this outbreak of DENV-2 and the findings of a case-control study examining risk factors for DHF.

## Methods

### Ethics Statement

When evaluated by the Institutional Review Board of the CDC, this non-research activity was considered a component of the public health response to the dengue outbreak in St. Croix and thus did not require review. Data analysis was performed on an anonymized dataset.

### Surveillance

Routine surveillance in the USVI relies upon healthcare provider-initiated requests for dengue laboratory testing submitted to the DB. Each diagnostic specimen is accompanied by a CDC Dengue Case Investigation Form (DCIF). The DCIF is used to report basic demographic and pertinent clinical information. The majority of the suspected dengue cases are reported from the island's sole tertiary care hospital, the Governor Juan F. Luis Hospital and Medical Center (JFL). Located in the capital, this 130-bed healthcare facility services the entire island.

### Case definitions

A suspected dengue case was defined as a febrile illness clinically compatible with DF according to the treating physician. World Health Organization (WHO) criteria [1] were used to classify clinical cases of DHF as follows: (1) fever, or a recent history of fever of 2–7 days duration, accompanied by (2) thrombocytopenia (≤100,000 cells/mm^3^), (3) hemorrhage, and (4) objective evidence of plasma leakage (e.g. pleural effusion or ascites, an abnormally low serum albumin or protein, or hemoconcentration [defined as hematocrit values ≥20% above mean values for sex and age [9] or ≥20% increase in hematocrit over the stabilized value at discharge]).

### Laboratory testing

All laboratory testing was performed at the DB. For laboratory diagnosis of dengue infections, a real-time, reverse transcriptase PCR assay (RT-PCR, TaqMan Applied Biosystems, Foster City, California, USA ) [10] or viral culture using C6/36 *Aedes albopictus* mosquito cells or tissues from intrathoracically-inoculated adult *Toxorhynchites amboinensis* mosquitoes [11], [12] was used to identify virus in serum specimens collected ≤5 days from the onset of fever (acute-phase serum specimen). To determine the outbreak strain, RNA was extracted from tissue culture supernatant (Qiagen). cDNA was generated using Sensiscript RT-PCR (Qiagen) with random hexamers (Applied Biosciences). Fourteen pooled overlapping 2000 nucleotide (nt) amplicons were generated by RT-PCR at the DB and sequenced at the Broad Institute by bidirectional Sanger. [13]

An immunoglobulin M (IgM) antibody-capture enzyme-linked immunosorbent assay (MAC-ELISA) was also used to detect anti-dengue IgM antibodies in acute and convalescent-phase (collected >5 days after the onset of fever) serum specimens. [14] If sufficient anti-dengue IgM antibodies were detected such that the optical density was ≥0.20, [14] this was considered evidence of a recent dengue infection. Serum specimens were also tested for the presence of anti-dengue immunoglobulin G (IgG) antibodies using an IgG ELISA. [15] The presence of anti-dengue IgG antibodies in acute serum specimens was used to distinguish primary (first) from secondary (sequential) dengue infections. An IgG titer ≥163,840 in a single serum specimen was also considered laboratory-positive for recent, secondary dengue infections even without positive virus identification results. [15] There was a limited supply of dengue antigen in 2005, thus a randomly selected subset of specimens were tested by IgG ELISA. All suspected dengue cases with laboratory evidence of an acute or recent infection were considered laboratory-positive. All suspected dengue cases with negative acute-phase results and no convalescent specimen were classified as indeterminate. All convalescent specimens that lacked an IgM response were laboratory-negative.

### Case-control study

To investigate risk factors for progression to DHF, we conducted a case-control study among laboratory-positive patients presenting to the Emergency Department (ED) or admitted to JFL Hospital during 2005. A case was defined as a laboratory-positive patient meeting all four WHO criteria for DHF. A control was defined as a laboratory-positive patient who did not meet criteria for DHF but was evaluated for thrombocytopenia, plasma leakage and hemorrhage. Hospitalized patients with negative or indeterminate dengue laboratory results were excluded from this analysis. The medical records of all cases and controls were reviewed. Data abstracted from the medical records included demographic information (date of birth, self-reported race on medical records, etc.) and information on clinical course and outcome. The range of normal values set by the hospital's clinical laboratory was used to define the threshold values for low serum albumin and protein levels.

### Statistical analysis

Data from the case-control study were entered directly into an electronic form designed using Questionnaire Development System v2.3 (Nova Research, Bethesda, MD, USA). All data analysis was then performed using SPSS version 12.0 (SPSS Inc, Chicago IL, USA) and SAS version 9.1 (SAS Institute, Cary NC, USA). The Student's t, Pearson's chi-squared, Fisher's exact, and Mann-Whitney U tests were used to assess unadjusted associations between study factors and DHF. All variables with a *p*-value less than 0.15 in bivariate analysis were included in the final model. Race [16] was added *a priori* to the final model. Adjusted odds ratios (ORs) and 95% confidence intervals (CIs) were calculated using exact multivariable logistic regression. All tests for significance were performed at an α-level of 0.05.

## Results

### Description of Outbreak

**Figure 1:**
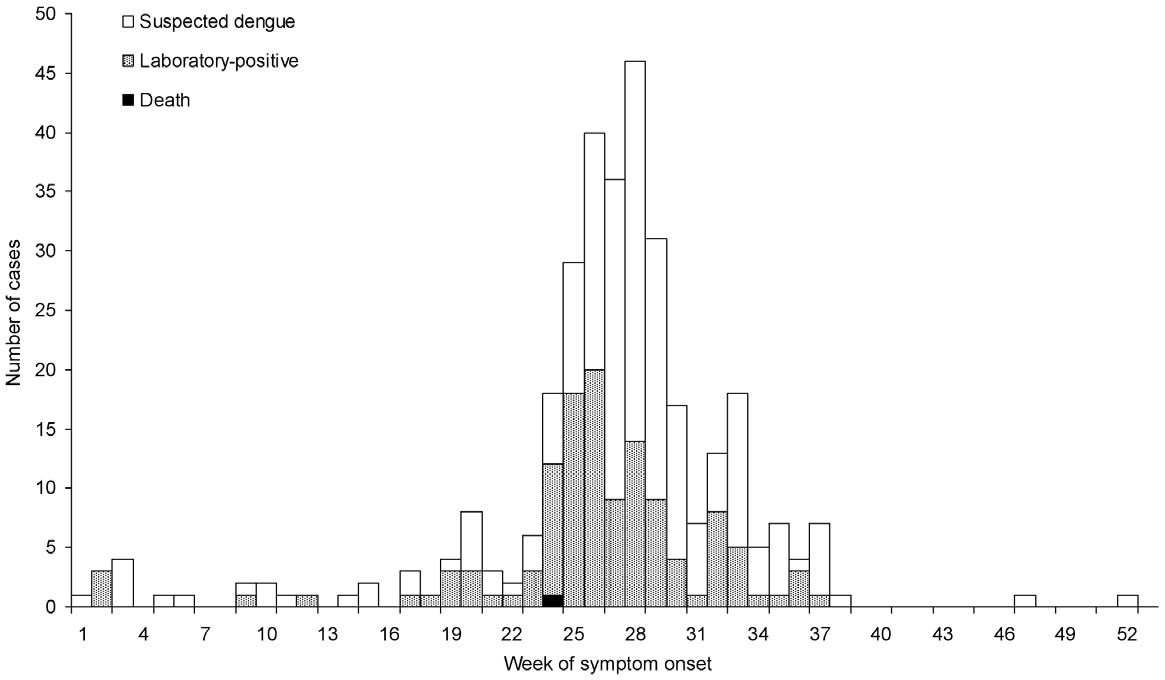
Number of suspected, laboratory-positive, and fatal dengue cases.

Between January 1 and December 31, 2005, 331 suspected DF cases (62.2 cases/10,000 population) were reported from St. Croix, USVI with a peak in July (Figure 1). Fifty-three percent were male, and the median age was 19 years (range: 3 months to 94 years). Cases were reported island-wide, but the most frequently reported place of residence (13%) was Williams Delight in southwest St. Croix. The most commonly reported signs and symptoms in suspected cases were headache (88%), fever (83%), body pain (83%), joint pain (68%), chills (56%), and retro-orbital pain (54%). Fifty-four percent of the suspected cases were hospitalized; 94 (28%) had thrombocytopenia, and 77 (21%) had at least one hemorrhagic manifestation.

Of the 331 suspected dengue cases, 123 (37%) were laboratory-positive, 32 (10%) were laboratory-negative, and 176 (53%) were indeterminate. The overall incidence of laboratory-positive cases was 23.1/10,000 population. Of the 44 laboratory-positive cases tested by the IgG ELISA, 3 (7%) were primary infections and 41 (93%) were secondary dengue infections. Fifteen (5%) of the suspected cases were laboratory-positive and met the WHO criteria for DHF. Of the 278 specimens tested by RT-PCR, 61 were identified as DENV-2. This was the only serotype identified from specimens collected during this outbreak. One of the DENV-2 isolates was sequenced and it corresponded to the southeast Asian lineage descending from the Jamaican 1983 isolate. [13]

One fatality occurred among the 331 suspected cases. The patient, a 14 year-old Afro-Caribbean female, presented to the hospital on day 3 post onset of illness with complaints of fever, generalized body pain and abdominal pain. Upon admission to the JFL Hospital, her platelet count was 33,000/mm^3^, and within the first 24 hours, she experienced hematuria, pleural effusions, severe abdominal pain, cold/clammy skin, and altered mental status. She suffered circulatory failure then expired soon thereafter. This patient met WHO criteria for DHF and was found to have had an acute, secondary dengue infection.

### Case-Control Study

**Figure 2:**
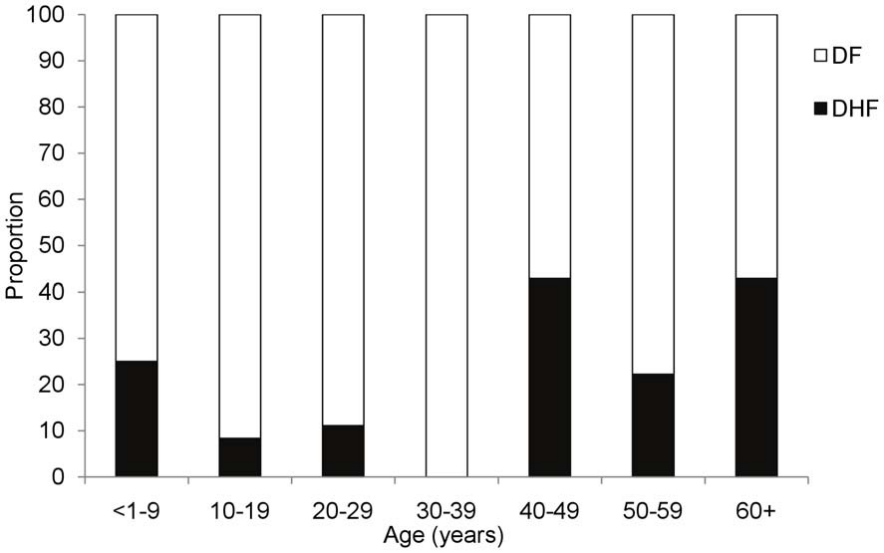
Proportion of DF and DHF cases in the case-control study by age-group.

Eighty-nine laboratory-positive patients were identified from the JFL Hospital including 15 (17%) who met WHO criteria for DHF (cases) and 74 (83%) who did not (controls). Of these 89 patients, 30% had at least one hemorrhagic manifestation. Of the 15 DHF cases, a single patient experienced DHF grade III and the remainder had grade II. [1] There was no significant difference in the median length of hospital stay between cases and controls (4.5 vs. 4.0 days, *p* = 0.74). Selected clinical characteristics of cases and controls are compared in Table 1. Cases and controls were similar with respect to gender, race, and history of hypertension, diabetes, or asthma. On bivariate analysis, age was the only variable significantly associated with DHF (*p* = 0.03). Similarly, in the final multivariable model (Table 2), age was the only variable associated with DHF (OR [95% CI]: 1.033 [1.003,1.064]). As age was considered as a continuous variable in the model, its OR is interpreted as follows: for every additional year of life, there would be a 3% increase in the odds of acquiring DHF. As demonstrated in table 3 and figure 2, there was a higher proportion of DHF cases in those above the age of 40.

**Table 1:** Bivariate associations between selected factors and DHF.

	Casen = 15	Controln = 74	*p*-value
Age (mean/years)	36	24	0.03
Male (%)	60	50	0.48
Afro-Caribbean (%)	64	72	0.75
History of hypertension (%)	7	8	1.00
History of diabetes (%)	7	5	1.00
History of asthma (%)	0	8	0.58
Experienced severe abdominal pain (%)	13	1	0.08
Experienced an abrupt change in temperature (%)	0	3	1.00

**Table 2:** Multivariable associations with DHF.

	Adjusted OR (95% CI)	*p*-value
Age/years	1.033 (1.003,1.064)	0.03
Afro-Caribbean	0.668 (0.185,2.406)	0.54
Experienced severe abdominal pain	9.542 (0.505,180.410)	0.13

**Table 3:** Frequency of cases and controls by age-group.

Age/years	<1–9	10–19	20–29	30–39	40–49	50–59	≥60
DF	6	33	16	3	4	7	4
DHF	2	3	2	0	3	2	3
Total	8	36	18	3	7	9	7

## Discussion

An outbreak of dengue fever occurred in the Caribbean island of St. Croix, USVI during June 2005. This is the largest recorded dengue outbreak to date in St. Croix and a high incidence of laboratory-positive infections was observed. In comparison to the recent large island-wide hyperendemic dengue outbreak in Puerto Rico, [16] the incidence observed in this outbreak was 2.7 times greater. Additionally, a high proportion of suspected cases (5%) met WHO criteria for DHF. In comparison, during the 1981 DENV-2 outbreak in Cuba, the proportion of DHF among reported cases was approximately 3%. [4] More recently, the proportion of DHF among reported dengue cases during the hyperendemic outbreaks of 2007 in the Caribbean states of Martinique, Guadeloupe, and the Dominican Republic similarly ranged from 2–3%. [17]

In this investigation, the enhancement of dengue surveillance at the hospital in St. Croix may have accounted for the increased proportion of DHF. It may also be explained by the high proportion of secondary infections, [2] as well as by the virulence of the circulating strain of dengue. [3] During this outbreak, the circulating dengue genotype was the southeast Asian strain of DENV-2, which has been previously demonstrated to be associated with more severe illness. [18]

To our knowledge, this is the first report of an investigation of the risk factors of DHF in the USVI. In this investigation, age was the only factor associated with DHF. The higher proportion of DHF cases in those above the age of 40 suggests that more of these individuals may have preexisting immunity to another dengue serotype after a past outbreak in St. Croix. An association between DHF and age has also been demonstrated using data from Brazil and Thailand. [19], [20] Additionally, there was a protective trend for Afro-Caribbean race in this study, but this was not statistically significant. Previous studies have noted that, despite hyperendemic transmission, DHF is not as prevalent in Haiti (where the majority of the population is of African descent) or in the African continent when compared to other tropical areas. [21], [22] This may be partially explained by the lack of consistent surveillance or by the under-diagnosis of DHF in these areas. In the Cuban dengue epidemics of 1981 and 2001, Afro-Cubans were at a reduced risk of severe manifestations of dengue when compared to whites. [23]–[25] More recently, it was reported that Afro-Colombians, in comparison with Mestizos, were less likely to be hospitalized, had higher platelet counts and lower hematocrit levels. [26] African ancestry has also been reported to be protective for DHF in Brazil. [27] Other studies have suggested that, in addition to race, there are other genetic determinants for severe dengue, ranging from blood group to expression of human leukocyte antigen (HLA) class I and II molecules on virus-infected cells. [28]–[31]

It is very difficult to assess race, and there are inconsistencies between different studies (*e.g*., researchers in Brazil used genetic ancestry as a proxy for race/ethnicity [27] while others in Colombia determined it by observing the hair type, facial features and skin color of study participants [26]). This study utilized self-reported race and, though an imperfect measure, this may be the most appropriate means of assigning it, [32] especially in the absence of ancestral genetic markers.

The major limitation of this investigation is the small sample size and consequent lack of power to detect significant associations. Moreover, 53% of suspected cases lacked a convalescent serum specimen and thus yielded indeterminate results from laboratory testing. Thus, in light of the small population size of St. Croix and the fact that only 37% of suspected cases were confirmed, any epidemiologic study on laboratory-positive cases seeking care at the hospital would be limited by a small sample size. There were inadequate resources to perform IgG testing on all specimens; thus the primary/secondary infection status of all cases and controls could not be ascertained, and this could not be assessed as a risk factor for DHF. However, increasing age may have acted as a proxy for secondary infection status. Additionally, a dengue seroprevalence study was not performed in response to this outbreak and the true incidence rate of dengue infection could not be calculated.

Severe dengue disease does occur along a spectrum but clinical data was insufficient for the non-hospitalized cases and inaccessible for those who sought care from private physicians during this outbreak. Thus, comparisons between severe and mild dengue cases were not possible. Other limitations include the high degree of under-reporting due to the passive method of dengue surveillance in the USVI. As all dengue testing is conducted outside of the territory, this may act as a further disincentive to report. Being unable to include data on laboratory-positive outpatients with dengue fever and the limited sample size and power of the study may have biased the results of this analysis towards the null. Conversely, the major strength of the case-control study was that it focused entirely on hospital-based, laboratory-positive patients which, through chart review, allowed for appropriate classification of disease severity.

This investigation provided an excellent opportunity to investigate the epidemiology of a circumscribed dengue outbreak in a Caribbean island – despite the increasing frequency of reported outbreaks in the region, there are few published reports documenting the unique nature of each outbreak. This is the first report addressing risk factors for DHF in the USVI, a multi-ethnic Caribbean territory. While it has been reported that persons of African descent are at reduced risk of DHF, clearly, as reported here, the population is still at risk of severe dengue and potentially death. HLA and other markers associated with altered risk of DHF have been identified, but the reason for this reduced risk among Afro-Caribbean persons remains elusive. The Caribbean is a dengue-endemic region and investigating outbreaks in its more populous multi-ethnic countries (such as Trinidad and Tobago, Cuba, and the Dominican Republic) would provide an excellent opportunity to conduct further research on the question of a dengue resistance gene in those of African descent.
